# 3D vs. 2D cephalometric analysis comparisons with repeated measurements from 20 Thai males and 20 Thai females

**DOI:** 10.2349/biij.5.4.e21

**Published:** 2009-10-01

**Authors:** W Bholsithi, W Tharanon, K Chintakanon, R Komolpis, C Sinthanayothin

**Affiliations:** 1 National Electronic and Computer Technology Center (NECTEC), National Science and Technology Development Agency (NSTDA), Pathumthani, Thailand; 2 Advanced Dental Technology Center (ADTEC), National Science and Technology Development Agency (NSTDA), Pathumthani, Thailand

**Keywords:** 3D cephalometric analysis, Simplant, cone-beam CT

## Abstract

This paper presented 3D cephalometric analysis on DICOM data from I-CAT CT cone-beam machine consisted of averages and standard deviations from 20 Thai males from 19 to 70 year (average 33.53 ± 14.08 year) and 20 Thai females from 16 to 70 year (average 32.60 ± 15.37 year). The angular measurements consisted of 49 lateral angular measurements and 9 frontal angular measurements while linear measurements consisted of 29 lateral linear measurements, 3 frontal linear measurements, and 8 perpendicular measurements. Results in 3D were compared with the corresponding 2D results showing that most midline-to-midline linear measurements and some midline-to-midline angular measurements were not different, while other types of measurements were significantly different. The 3D results will be used in the clinical Ceph3D services as requested by those with interests on cephalometric analysis and anthropology with focus on Thai subjects while the 2D results will be used for comparison with cephalometric analyses from other orthodontists.ts reserved.

## INTRODUCTION

Cephalometric analysis is one of the essential tools in orthodontic diagnoses as well as craniomaxillofacial surgery. Two-dimensional cephalometric measurements from lateral and/or frontal cephalograms were widely studied in several ethnic groups [1][2] including Thai people [3].

However, 2D-cephalometry is a projection image of 3D-structures, which has several disadvantages including non-homogenous enlargement and distortion on lateral structures, inaccurate landmark locations due to overlapping structures, and landmarks that appear on the lateral may not appear on the frontal image or vice versa. Misaligned head position may lead to fault diagnosis.

In addition, using average measurements of left and right structures in 2D-cephalometry as though both sides of the face are symmetrical is not realistic since human face is rarely symmetrical [4]. Olszewski et al. has demonstrated that 3D analysis gives the same results and adequate diagnoses as 2D analysis using the same skull [5] while Adam et al. has shown that using a 3D method is more precise with 4-5 times more accurate than the 2D approach [6]. However, a few 3D cephalometric analysis researches were focusing on a large number of samples [7-8] including Thai cephalometric researches [9-10] but most of them did not take landmarks on facial soft tissue into account.

## METHODOLOGY

### Hardware and Software

I-CAT cone beam CT scan was used with 512 x 512 matrices, radiation at 120 kV and 87.75 mAs taken at 0.4 mm slice thickness. Simplant Master^TM^ (Materialise N.V.), medical image processing software, was used for 3D reconstruction from CT DICOM data with 0.4 mm interpolated slice thickness. All anatomical landmarks were first identified on the 3D model, and their positions were verified in multi-planar reformat mode in axial and sagittal views.

The selected means and standard deviations plots of thirty eight landmark positions from repeated tests can be classified according to craniofacial landmarks types including 5 Anterior Cranial based, 5 Nasomaxillary Complex, 10 Mandible, 14 Dentition, and 4 Soft tissue to be listed in details as follows:

1. Anterior Cranial Based Landmarks including Nasal (N), Sella (S), Left Porion (PoL), and Right Porion (PoR)
2. Nasomaxillofacial complex landmarks including Subspinal (A), Anterior Nasal Spine (ANS), Posterior Nasal Spine (PNS), Basion (Ba), Left Orbitale (OrL), and Right Orbitale (OrR)
3. Mandible landmarks including Left Gonion (GoL), Right Gonion (GoR), Left Condyle Head (CondL), Right Condyle Head (CondR), Center of Left Condyle (CcL), Center of Right Condyle (CcR), Subspinal (B), Pogonion (Pog), Menton (Me), and Gnathion (Gn)
4. Dentition Landmarks including Upper left incisor tip (A1L), Upper right incisor tip (A1R), Upper left incisor apex (ARL),Upper right incisor apex (ARR), Lower left incisor tip (B1L), Lower right incisor tip (B1R), Lower left incisor apex (BRL), Lower right incisor apex (BRR), Upper left Canine tip (A3L), Upper right Canine tip (A3L), Lower left Canine tip (B3L),Lower right Canine tip (B3R), First Buccal of the first Left Molar (B6L), and First Buccal of the first Right Molar (B6R)
5. Soft Tissue Landmarks including Pronasale (PRN), Labial Superior (Ls), Labial Inferior (Li), and Soft Tissue Pogonion (PG)

Fifty-eight angular measurements, forty linear measurements, and a ratio [11-14] based upon thirty-eight landmarks were analyzed from CT radiographs of 20 men and 20 women, non-severe malocclusion Thai patients. The ages of 20 males were ranged from 19 to 70 years with the mean of 33.53 ± 14.08 years while the ages of 20 female patients were ranged from 16 to 70 years with the mean of 32.60 ± 15.37 years.

Linear measurements consisted of 31 lateral linear measurements including 9 midline-to-midline, and 22 lateral-to-lateral, 3 frontal and 8 perpendicular linear measurements to be listed along with the analysis results in Table 1.

**Table 1:** Linear cephalometric results from male and female samples.

**Types of Linear Measurement**	**Linear Measurements (Degrees)**	**Men**				**Female**				**P M-F 3D**	**P M-F 2D**
		**Mean ± SD 3D**	**Mean ± SD 2D**	**% Diff**	**P 3D – 2D**	**Mean ± SD 3D**	**Mean ± SD 2D**	**% Diff**	**P 3D – 2D**		
**Midline To Midline**	A-B	34.20 ± 0.52	34.19 ± 0.52	0.88	NS	40.56 ± 5.33	40.94 ± 6.75	0.92	NS	NS	NS
	ANS-Me	65.34 ± 0.20	65.34 ± 0.20	0.98	NS	69.01 ± 6.98	68.29 ± 9.81	1.05	NS	NS	NS
	N-ANS	54.24 ± 4.79	54.24 ± 4.79	0.68	NS	49.77 ± 3.32	50.10 ± 5.69	0.66	NS	**	NS
	S-ANS	84.95 ± 0.16	84.84 ± 0.22	0.88	NS	81.53 ± 4.74	78.52 ± 11.8	3.84	NS	***	*
	S-N	66.51 ± 0.78	66.36 ± 0.84	1.72	NS	64.77 ± 3.12	60.96 ± 13.9	6.25	NS	***	*
	ANS-A1L	29.83 ± 2.67	29.40 ± 2.68	0.24	***	30.00 ± 3.70	29.45 ± 3.84	1.85	*	NS	NS
	ANS-A1R	29.95 ± 2.67	30.02 ± 4.29	1.52	NS	30.12 ± 3.68	30.13 ± 5.25	0.05	NS	NS	NS
	Me-B1L	43.20 ± 2.92	43.10 ± 2.93	1.46	***	42.24 ± 4.05	42.08 ± 4.06	0.38	***	NS	NS
	Me-B1R	43.09 ± 2.86	43.76 ± 7.79	0.23	NS	42.27 ± 3.93	42.78 ± 8.22	1.20	NS	NS	NS
**Lateral to Lateral**	A-OrL	49.90 ± 2.91	34.04 ± 2.95	46.59	***	46.66 ± 3.63	44.44 ± 3.04	5.01	***	***	***
	A-OrR	49.49 ± 2.74	33.91 ± 3.01	45.97	***	46.78 ± 3.27	43.93 ± 2.92	6.49	***	*	***
	A – CondL	98.45 ± 4.31	83.24 ± 5.45	18.27	***	94.22 ± 4.50	78.23 ± 8.17	20.45	***	**	**
	A – CondR	98.73 ± 3.96	84.17 ± 7.19	17.30	***	94.56 ± 4.39	79.84 ± 10.4	18.45	***	**	NS
	A-CcL	98.20 ± 3.96	82.08 ± 4.56	19.64	***	93.71 ± 4.68	55.44 ± 3.66	69.02	***	**	***
	A-CcR	98.48 ± 3.93	82.58 ± 8.46	19.25	***	94.09 ± 4.35	52.10 ± 5.19	80.60	***	**	***
	CcL-GoL	55.08 ± 5.70	54.25 ± 7.68	1.53	NS	52.53 ± 5.11	51.59 ± 7.09	1.82	NS	NS	NS
	CcR-GoR	54.93 ± 5.83	55.09 ± 7.95	0.29	NS	52.63 ± 5.57	52.67 ± 8.54	0.08	NS	NS	NS
	Gn – CondL	128.49 ± 6.70	117.20 ± 6.86	9.63	***	124.85 ± 6.50	113.15 ± 8.12	10.34	***	*	*
	Gn – CondR	128.97 ± 6.92	116.58 ± 14.1	10.63	***	125.52 ± 6.65	113.01 ± 14.2	11.07	***	*	NS
	Me-CcL	123.64 ± 6.45	72.77 ± 7.24	5.49	***	120.09 ± 6.09	107.47 ± 7.72	11.75	***	*	***
	Me-CcR	124.03 ± 6.60	74.07 ± 7.65	6.39	***	120.67 ± 6.31	108.12 ± 9.42	11.60	***	*	***
	Me-GoL	88.21 ± 4.30	95.94 ± 5.22	21.22	***	86.12 ± 4.57	70.73 ± 7.89	21.77	***	*	***
	Me-GoR	88.86 ± 4.27	95.20 ± 6.20	19.96	***	86.29 ± 4.69	72.12 ± 8.88	19.66	***	**	***
	Me-OrL	102.51 ± 5.27	111.46 ± 6.80	6.85	***	98.51 ± 7.36	92.87 ± 6.86	6.07	***	*	***
	Me-OrR	102.28 ± 4.96	111.59 ± 9.23	7.43	***	98.63 ± 7.19	91.90 ± 8.29	7.32	***	*	***
	Pog – CondL	126.60 ± 6.76	115.11 ± 7.00	9.98	***	122.99 ± 6.41	110.89 ± 8.33	10.91	***	*	*
	Pog - CondR	127.07 ± 6.99	115.58 ± 8.57	9.94	***	123.74 ± 6.49	111.99 ± 9.29	10.49	***	*	NS
	B1L – CcL	107.49 ± 4.78	93.64 ± 10.9	14.78	***	103.74 ± 4.92	88.48 ± 12.2	17.25	***	**	*
	B1R – CcR	108.12 ± 5.13	94.54 ± 12.0	14.37	***	104.17 ± 4.72	89.69 ± 14.0	16.15	***	*	NS
	A1L – OrL	66.98 ± 3.26	58.49 ± 6.67	14.52	***	63.67 ± 5.58	56.03 ± 7.41	13.64	***	NS	NS
	A1R – OrR	67.17 ± 3.47	58.87 ± 4.50	14.11	***	64.21 ± 5.06	56.19 ± 6.20	14.27	***	**	NS
**Frontal Left to Right**	CcR – CcL	108.06 ± 1.11	107.78 ± 1.10	0.10	***	101.43 ± 6.14	101.21 ± 6.19	0.22	*	**	**
	GoR – GoL	105.95 ± 0.65	105.56 ± 0.67	0.21	**	90.89 ± 6.08	90.70 ± 6.13	0.21	**	**	**
	OrL-OrR	64.95 ± 1.22	64.75 ± 1.21	0.12	***	68.72 ± 6.30	68.75 ± 6.32	0.04	NS	*	*
**Perpendicular Distance**	*U1L-NA	9.66 ± 0.21	7.61 ± 0.24	48.87	***	7.41 ± 1.81	4.90 ± 2.30	51.28	***	NS	NS
	*U1R-NA	7.34 ± 0.32	7.06 ± 0.34	38.57	***	6.70 ± 2.02	4.71 ± 2.45	42.47	***	NS	NS
	*U1-NA	6.78 ± 1.51	4.71 ± 2.02	43.77	***	7.06 ± 1.77	4.80 ± 2.31	46.96	***	NS	NS
	*L1L-NB	6.73 ± 0.23	5.59 ± 0.21	9.98	***	6.73 ± 2.58	5.89 ± 2.96	14.18	**	NS	NS
	*L1R-NB	5.96 ± 0.53	5.80 ± 0.45	10.94	***	7.22 ± 2.51	6.38 ± 2.64	13.18	***	NS	NS
	*L1-NB	6.68 ± 1.86	6.05 ± 2.03	10.48	***	6.97 ± 2.49	6.13 ± 2.74	13.66	***	NS	NS
	UL to E-Line	0.89 ± 0.76	3.60E-07 ± 4.93E-07	16.10	***	2.91 ± 2.01	2.53 ± 2.15	15.16	**	NS	NS
	LL to E-Line	1.75 ± 0.85	0.87 ± 0.99	23.93	***	2.68 ± 1.75	2.32 ± 1.85	15.47	***	*	*
**Distance Ratio**	N-ANS/ANS-Me	83.01 ± 7.31	83.01 ± 7.31	0.03	NS	73.05 ± 9.92	72.84 ± 9.88	0.28	NS	NS	NS

Angular measurements consisted of 49 lateral angular measurements including 19 three or four points all midline, 10 one point midline and two point lateral, 6 midline-midline to midline-lateral four points, 4 midline-lateral to lateral-lateral four points, 8 midline-midline to lateral-lateral four points, 2 four point lateral, and 9 frontal angular measurements to be listed along with the analysis results in Table 2.

**Table 2:** Angular cephalometric results from male and female samples

**Types of Angular Measurement**	**Angular Measurements (Degrees)**	**Men**				**Women**				**P M- F 3D**	**P M- F 2D**
		**Mean ± SD 3D**	**Mean ± SD 2D**	**% Diff**	**P 3D – 2D**	**Mean ± SD 3D**	**Mean ± SD 2D**	**% Diff**	**P 3D – 2D**		
**Three or Four Points All Midline**	SNA	87.49 ± 3.57	86.72 ± 8.89	0.89	NS	86.89 ± 4.36	82.52 ± 17.9	5.30	NS	NS	NS
	SNB	84.07 ± 3.84	83.30 ± 8.68	0.93	NS	84.07 ± 3.44	79.44 ± 17.5	5.82	NS	NS	NS
	ANB	3.73 ± 2.04	3.52 ± 2.10	5.74	**	3.61 ± 2.23	3.54 ± 3.46	1.94	NS	NS	NS
	B1L to NB	4.95 ± 1.35	4.50 ± 1.45	10.00	***	5.43 ± 2.01	4.75 ± 2.29	14.51	**	NS	NS
	B1R to NB	5.01 ± 1.32	4.52 ± 1.46	10.96	***	5.34 ± 1.87	4.72 ± 1.96	12.97	***	NS	NS
	NSBa	123.21 ± 4.60	123.77 ± 6.71	0.45	NS	124.17 ± 5.94	125.68 ± 13.0	1.20	NS	NS	NS
	L1L to NB	34.11 ± 3.84	30.50 ± 5.07	11.84	***	33.64 ± 6.30	29.95 ± 6.77	12.33	***	NS	NS
	L1R to NB	30.68 ± 5.52	30.50 ± 5.58	0.57	***	30.21 ± 6.63	30.14 ± 6.67	0.23	NS	NS	NS
	L1L to SN	54.04 ± 6.51	53.95 ± 6.54	0.17	*	53.40 ± 8.87	53.43 ± 8.94	0.05	NS	NS	NS
	L1R to SN	53.68 ± 7.25	53.58 ± 7.29	0.17	**	54.11 ± 7.85	53.79 ± 8.33	0.60	NS	NS	NS
	U1L to ANS – PNS	65.31 ± 8.41	65.21 ± 8.41	0.17	**	64.48 ± 7.77	64.36 ± 7.72	0.19	*	NS	NS
	U1R to ANS – PNS	65.36 ± 8.68	65.36 ± 8.70	0.01	NS	65.32 ± 7.10	65.31 ± 7.11	0.01	NS	NS	NS
	U1L to NA	22.36 ± 7.44	21.68 ± 7.76	0.21	***	23.30 ± 8.03	22.78 ± 8.25	149.28	***	***	***
	U1R to NA	22.09 ± 7.18	21.39 ± 7.90	0.18	***	22.16 ± 7.80	21.86 ± 7.83	151.49	***	***	***
	U1L to L1L	124.71 ± 10.3	124.98 ± 10.4	3.13	*	56.77 ± 11.5	56.15 ± 12.0	58.50	***	NS	***
	U1R to L1R	124.51 ± 10.1	124.74 ± 10.3	3.29	*	54.96 ± 10.0	54.45 ± 10.2	59.30	***	NS	***
	U1L to SN	70.93 ± 7.88	70.85 ± 7.91	0.12	*	70.24 ± 7.31	70.05 ± 7.36	0.27	*	NS	NS
	U1R to SN	71.11 ± 8.09	71.12 ± 8.08	0.01	NS	71.13 ± 6.73	71.23 ± 6.72	0.14	NS	NS	NS
	ANS-PNS to SN	6.48 ± 4.20	6.03 ± 4.50	7.56	***	6.63 ± 3.52	7.27 ± 9.80	8.77	NS	NS	NS
**One point midline to Two points lateral**	A to FHL	112.53 ± 3.04	101.30 ± 18.7	11.08	**	113.62 ± 3.04	111.84 ± 4.71	1.59	NS	NS	*
	A to FHR	113.23 ± 2.72	100.35 ± 18.6	12.83	**	113.79 ± 3.71	112.84 ± 11.1	0.84	NS	NS	**
	Me to GoL to CcL	117.72 ± 6.58	120.19 ± 10.8	2.06	NS	118.13 ± 4.77	120.65 ± 6.96	2.09	***	NS	NS
	Me to GoR to CcR	117.61 ± 6.47	120.57 ± 11.6	2.45	NS	118.80 ± 5.67	120.52 ± 8.02	1.43	*	NS	NS
	Gn-GoL to CondL	114.36 ± 6.43	117.06 ± 7.22	2.30	***	114.74 ± 4.83	117.23 ± 7.44	2.12	**	NS	NS
	Gn-GoR to CondR	114.22 ± 5.90	116.31 ± 7.25	1.80	**	115.28 ± 5.81	116.90 ± 8.01	1.39	*	NS	NS
	L1L to FHL	60.24 ± 6.17	59.38 ± 6.62	1.45	***	61.18 ± 8.67	60.41 ± 9.19	1.27	*	NS	NS
	L1R to FHR	60.24 ± 7.46	59.44 ± 7.40	1.35	**	62.02 ± 7.27	61.25 ± 7.67	2.25	**	NS	NS
	U1L to FHL	66.64 ± 8.67	65.55 ± 8.92	1.66	***	64.84 ± 7.93	63.41 ± 7.97	2.25	***	NS	NS
	U1R to FHR	65.91 ± 8.48	65.30 ± 8.27	0.92	***	64.90 ± 6.77	64.21 ± 6.95	1.07	***	NS	NS
**Midline- Midline to midline lateral Four Points**	L1L to GoL-Gn	77.14 ± 4.52	82.58 ± 4.18	6.59	***	78.01 ± 7.40	81.79 ± 4.66	4.62	*	NS	NS
	L1R to GoR-Gn	83.40 ± 4.26	82.27 ± 4.92	1.37	***	83.72 ± 4.26	82.00 ± 5.53	2.10	**	NS	NS
	L1L to Me – GoL	85.42 ± 3.83	84.74 ± 4.63	0.79	***	82.77 ± 5.44	81.23 ± 7.88	1.90	*	*	*
	L1R to Me – GoR	84.46 ± 3.98	83.73 ± 4.80	0.87	*	84.07 ± 3.87	81.99 ± 7.43	2.54	*	NS	NS
	Me-GoL to SN	46.11 ± 4.83	34.04 ± 6.20	35.46	***	45.76 ± 4.22	35.32 ± 6.84	29.56	***	NS	NS
	Me-GoR to SN	45.95 ± 5.03	33.53 ± 6.53	37.05	***	45.38 ± 4.58	33.62 ± 6.53	34.98	***	NS	NS
**Midline- lateral to lateral- lateral Four points**	Gn - GoL to POPL	19.66 ± 3.66	22.06 ± 4.83	10.85	***	20.07 ± 5.02	22.96 ± 6.34	12.61	***	NS	NS
	Gn - GoR to POPR	20.44 ± 4.36	22.50 ± 5.36	9.16	***	20.71 ± 5.33	22.30 ± 6.53	7.14	***	NS	NS
	Me-GoL to FHL	35.17 ± 4.62	28.09 ± 6.44	23.12	***	32.77 ± 5.41	28.09 ± 6.44	16.67	***	NS	NS
	Me-GoR to FHR	34.39 ± 4.37	27.48 ± 10.0	24.54	***	32.79 ± 5.59	27.48 ± 10.0	19.35	***	NS	NS
**Midline- midline to lateral- lateral Four points**	ANS-PNS to FHL	10.63 ± 4.08	2.35 ± 2.25	233.93	***	12.12 ± 3.19	2.35 ± 2.25	415.60	***	NS	NS
	ANS-PNS to FHR	11.23 ± 3.60	2.33 ± 2.15	284.45	***	11.63 ± 3.02	2.33 ± 2.15	399.94	***	NS	NS
	SN to POPL	30.57 ± 4.93	9.79 ± 5.60	228.97	***	30.99 ± 4.57	9.79 ± 5.60	216.48	***	NS	NS
	SN to POPR	30.13 ± 5.28	10.21 ± 13.1	249.46	***	29.31 ± 4.68	10.21 ± 13.1	186.96	***	NS	NS
	SN to FHL	11.68 ± 4.00	6.91 ± 3.13	113.09	***	13.84 ± 4.01	6.91 ± 3.13	100.20	***	*	NS
	SN to FHR	12.21 ± 4.00	7.28 ± 3.64	105.94	***	13.30 ± 3.78	7.28 ± 3.64	82.61	***	NS	NS
	SN to GoL – Gn	43.27 ± 4.71	31.20 ± 5.80	38.69	***	43.04 ± 4.12	32.07 ± 5.30	34.21	***	NS	NS
	SN to GoR – Gn	43.21 ± 4.79	30.79 ± 6.10	40.36	***	42.75 ± 4.39	31.00 ± 6.05	37.89	***	NS	NS
**Four point Lateral**	FHL to POPL	19.93 ± 5.15	5.52 ± 4.51	261.04	***	18.43 ± 5.34	5.40 ± 3.71	241.27	***	NS	NS
	FHR to POPR	19.12 ± 5.03	5.04 ± 4.10	279.67	***	17.44 ± 5.57	4.98 ± 3.31	249.98	***	NS	NS
**Frontal Analysis**	CcR to A to CcL	65.72 ± 3.74	153.27 ± 13.5	57.12	***	65.42 ± 3.17	145.47 ± 18.3	55.02	***	NS	NS
	CcR to B1L to CcL	58.62 ± 4.14	115.41 ± 17.5	49.20	***	57.58 ± 3.36	105.97 ± 13.5	45.67	***	NS	NS
	CcR to B1R to CcL	58.58 ± 4.11	115.20 ± 17.4	49.15	***	57.70 ± 3.29	105.75 ± 13.4	45.44	***	NS	NS
	CcR to Me to CcL	51.13 ± 3.91	70.35 ± 9.10	27.32	***	49.913 ± 3.30	65.22 ± 7.04	23.48	***	NS	*
	GoR to Me to GoL	66.51 ± 5.14	128.22 ± 17.0	48.12	***	63.69 ± 3.81	117.18 ± 15.8	45.65	***	NS	*
	OrR to A to OrL	94.00 ± 6.14	104.07 ± 9.54	9.68	***	94.79 ± 6.09	102.35 ± 7.62	7.39	***	NS	**
	OrR to Me to OrL	41.59 ± 3.22	42.45 ± 4.06	2.03	***	40.83 ± 2.76	41.29 ± 2.95	1.13	***	*	**
	GoR-GoL to AO	3.42 ± 1.99	36.62 ± 26.9	90.65	***	3.19 ± 1.53	39.04 ± 25.7	91.82	***	NS	NS
	OrR-OrL to AO	2.94 ± 1.69	34.14 ± 29.3	91.38	*	3.03 ±1.90	35.89 ± 25.4	91.54	***	NS	NS

**Figure 1:**
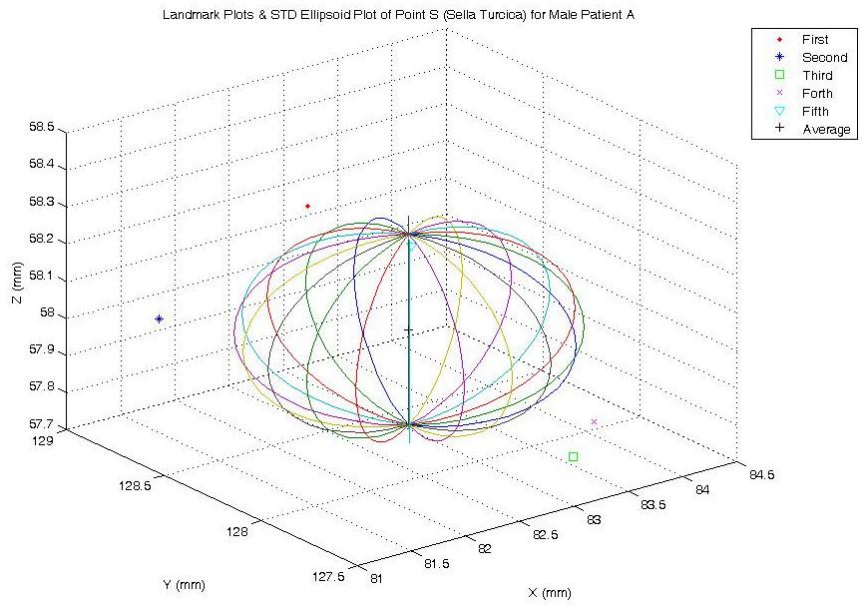
2D and 3D cephalometric measurements. (a) 3D Lateral, (b) 3D Frontal, (c) 2D Lateral, and (d) 2D Frontal.

Fig 1a and 1b depicted 3D images where 3D cephalometric analysis was derived from Simplant CMF^TM^ was applied to calculate default 2D cephalometric analysis in form of lateral x-ray in Fig 1c. Applying sagittal plane readjustment to display an x-ray image of frontal skull and get 2D frontal analysis as shown in Fig 1d. Subsequently, 3D cephalometric analysis was compared with corresponding 2D lateral and frontal analysis.

### Analyses and Calculations

Data of 20 males and 20 females were digitized and had landmarks located five times by the same operator for the test of accuracy and reliability. Dahlberg’s formula of standard errors was applied to analyze the positions of 38 landmarks as applied in the work of Hashim [15] which is the square of different between mean position and actual results on x, y, and z axis.

(1) $$D=\sqrt{\frac{\sum_{i=1}^{5}d_{i}^{2}}{2n}}$$

**Figure 2:**
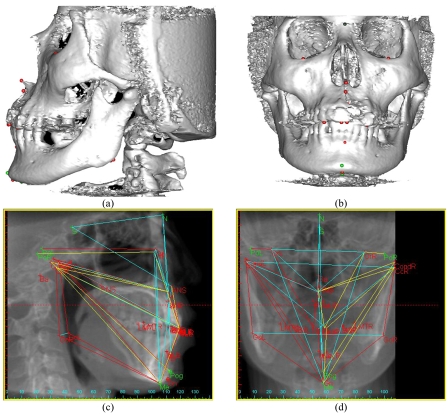
The result from repeated tests on sella turcica (Point S).

The means and standard deviations of landmark positions on x, y, and z axis will be plotted as ellipsoid along with and a set of 5 landmarks from repeated tests by using MATLAB® as shown for the case of Sella Turcica (Point S) in Figure 2. After obtaining the linear and angular measurements, paired T-Test through command TTEST of Microsoft Excel® was used to analyze the differences between 3D and 2D measurements and the differences between 2D and 3D measurements from male examples and the correspondent measurements from female examples at p<0.05. The differences were shown in percentage using the formula with the results rounded to integers.

(2) $$Percent=\frac{\left|3D-2D\right|}{2D}x100$$

The paired T-Test results will be shown as the probabilities to be described as follows: NS is for non significant for the case with probability over 0.05 which implied that the pair of analyzed values is interchangeable while * is for the case with probability less than 0.05 (p< 0.05), ** is for the case with probability less than 0.01 (p< 0.01), and *** is for the case with probability less than 0.001 (p< 0.001) which implied that the pair of analyzed values is not interchangeable.

## RESULTS

The repeated test results of 38 landmarks in males showed that the highest errors on x-axis were at PNS due to difficulties to pinpoint the back end of palate (PNS) to be accurate in all axes simultaneously. The errors on Y-axis occurred at the highest level at the buccal of the first right molar (B6R) as well as the left and right gonion (GoL and GoR) due to the radiographic scattering from the filling that blur both CT images and rendered 3D images, and The highest error on Z axis were the upper lip (Ls) and lower lip (Li) due to the difficulties to pinpoint the position of these 2 soft tissue landmarks which required an observer to view both sagittal and lateral projection simultaneous as a counter-check measure for 3D landmarking.

The repeated test results of 38 landmarks in females showed that the highest errors on x- axis were at the upper end of right porion (PoR) due to the limited field of view (FOV). The errors on Y-axis occurred at the highest level at the buccal of first right molar (B6R) due to the radiographic scattering from the filling that blurs both CT images and rendered 3D images, and the highest error on Z axis were the subspinal (B), center of right condyle (CcR), lower lip (Li) and soft tissue pogonion (PG) due to the difficulties to pinpoint the position of these landmarks which required an observer to view both sagittal and lateral projection simultaneous as a countercheck measure for 3D landmarking.

The paired T-test results of linear measurements from 20 males and 20 female along with 2D and 3D comparison were shown in Table 1. Linear measurement from 20 males showed that most measurements from midline to midline structures were not significantly different between 3D and 2D cephalometry as well as N-ANS/ANS-Me ratio while the other types of measurements were significantly different. Furthermore, results from 20 males implied that 2D linear measurements can be substituted by the corresponding 3D linear measurements in most of midline to midline cases and a few measurements of lateral to lateral and N-ANS/ANS-Me ratio.

Results for corresponding linear measurement from 20 females in Table 1 also showed similar results as male counterparts with noticeable differences in OrL – OrR, Gn – CondL, and Gn – CondR showing that 3D and 3D linear measurements can be substitute for male cases but not substitutable in female cases and vice versa.

Linear measurement comparisons in Table 1 showed that linear measurements from male samples are generally different from the corresponding linear measurements from female samples, and the 3D linear measurements are showing larger differences than the corresponding 2D linear measurements so few 3D linear measurements from male samples are interchangeable with the corresponding 3D linear measurements from female samples. The exceptions are the linear measurements on perpendicular distances that show much smaller differences between 2D and 3D linear measurements; therefore, most of perpendicular distances from male samples can be interchanged with the corresponding perpendicular distances from female samples.

The paired T-test results of angular measurements from 20 males and from 20 females along with angular measurements comparisons were shown in Table 2

Results from 20 males implied that few 2D angular measurements including SNA, SNB, NSBa, U1R to ANS-PNS, U1R to SN, Me to GoL to CcL, Me to GoR to CcR could be substituted by the corresponding 3D angular measurements while the other angular measurements could not.

Results from the 20 females also showed similar results as male counterparts with the additional 2D angular measurement which can be substituted by the corresponding 3D angular measurements including ANB, L1R to NB, L1L to SN, L1R to SN, ANS – PNS to SN, A to FHL, and A to FHR.

Angular measurement comparisons showed that most of 2D and 3D angular measurements from male examples could be interchanged with the correspondent angular measurements from female examples. However, the differences were the interincisal angles (U1L-L1L, U1R-L1R) which show that the 3D measures from males can be inter-changed with the corresponding results from females but not interchangeable for the case of 2D angular measurements.

## DISCUSSION

**Figure 3:**
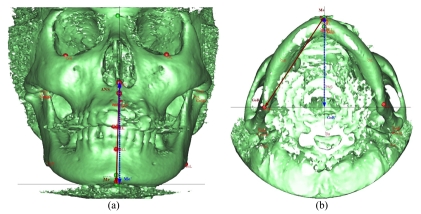
The Diagrams showing differences between 3D and 2D linear measurements.

The comparison of 3D and 2D linear measurements derived from midline structure to midline structure (e.g. A-B, ANS-Me) and measurements derived from lateral structure to lateral structure (e.g. CcL-GoL, CcR-GoR) as the example to the measurement of lower face height in Figure 3a with ANS-Me as the 3D measurement of lower face height and ANS-Me’ as the 2D measurement of lower face height. However, all 3D measurements derived from midline structures to lateral structures were larger than those of 2D because 2D measurements were projected image rather than true measurement. Fig. 3b shows that Me-GoR represents the right mandibular length in 3D while Me-GoR’ represents the corresponding distance in 2D.

**Figure 4:**
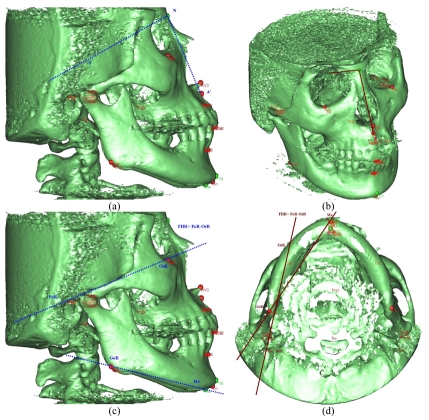
Diagrams showing differences between 3D and 2D angular measurements.

Angular measurements derived from all the landmarks in mid-sagittal plane (e.g. SNA, SNB) showed similar results between 3D and 2D to the level that it can be substituted as shown the measurement of sagittal maxillary position in Fig. 4a and 4b with Fig. 4a shows that SNA’ represents the angular measurement of sagittal maxillary position in 2D while Fig. 4b shows that SNA represents the angular measurement of sagittal maxillary position in 3D. Angular measurements derived from 1point midline to 2 points lateral (e.g. A to FHL, A to FHR) in 3D showed minor differences from 2D measurements. However, measurements derived from 4 points in different planes, 3D and 2D data had significant differences since measurements in 3D were not measured from the same projected planes as in 2D so angular measurements in 3D should not be interpreted in the same way as conventional 2D. Diagrams in Fig. 4c and (D) show different results between 3D and 2D measurement of the right mandibular height, the angle between right mandibular length (Me-GoR) and right Frankfort Horizontal plane (FHR) which is the plane through right porion and right orbitale (PoR-OrR). Fig. 4c shows projected measurement from 2D onto mid-sagittal plane and Fig. 4d shows that GoR and FHR are not on the same plane in space.

Landmarks such as left and right porion (PoL, PoR) along with left and right condylion (CondL, CondR) were difficult to locate due to the narrow field of view of the CT scan that was too small to cover these landmarks in patients with big skulls. In general, the standard deviations of most measurements in this study were higher than previous studies [7-9] due to the data collected from patient group, which have larger variation than the data collected from population with normal occlusion.

## CONCLUSIONS

The results from Ceph3D analyses will be applied in the clinical Ceph3D services as requested by those with interests on cephalometric analysis and anthropology with focus on Thai subjects while the 2D results will be used for comparison with cephalometric analyses from other orthodontists. Nevertheless, the standard Ceph3D analyses were subjected for the further revisions to accommodate more types of measurements as well as more data from subjects.
